# The Effect of Frequency of Fresh Pasture Allocation on the Feeding Behaviour of High Production Dairy Cows

**DOI:** 10.3390/ani12030243

**Published:** 2022-01-20

**Authors:** Jessica G. Pollock, Alan W. Gordon, Kathryn M. Huson, Deborah A. McConnell

**Affiliations:** 1 School of Biological Sciences, Queens University Belfast, Belfast BT9 7BL, UK; 2 Agri-Food and Biosciences Institute, Large Park, Hillborough BT26 6DR, UK; kathryn.huson@afbini.gov.uk (K.M.H.); deborah.mcconnell@afbini.gov.uk (D.A.M.); 3 Agri-Food and Biosciences Institute, 18a Newforge Lane, Belfast BT9 5PX, UK; alan.gordon@afbini.gov.uk

**Keywords:** dairy cows, grazing management, animal behavior, grazing, ruminating, parity

## Abstract

**Simple Summary:**

In pasture based systems dairy cows spend more than 50% of their time grazing and ruminating, thus these behaviours require a lot of time and energy. Understanding the impact of management factors such as pasture allocation frequency on animal feeding behaviour will assist with the development of systems that support natural and efficient animal feeding behaviour. The aim of this study was to investigate the effect of frequency of fresh pasture allocation, three treatments offering fresh pasture every 12, 24 or 36 hours on the grazing and ruminating behaviours of high-yielding dairy cows. Animals displayed diurnal feeding patterns, irrespective of treatment, concentrating the majority of their grazing activity during the day (90%) and their ruminating activity during the night (73%). Peak grazing activity coincided with fresh pasture allocation in the 12 h and 24 h treatments. However, in the 36 h treatment peak grazing activity did not correspond with the allocation of fresh pasture and grazing was more evenly distributed over each 24 h period, indicating the animals’ inability to anticipate feed. Increased competition for resources in the 12 h treatment likely resulted in the greater grazing and ruminating times exhibited by primiparous animals, indicating greater overall energy expenditure on feeding behaviour.

**Abstract:**

For ruminants, grazing and ruminating activities are essential in nutrient capture and ultimately animal performance however these activities can demand significant time and energy. This study evaluated the effect of three different pasture allocation frequencies (PAF’s; 12, 24 and 36 h) on the feeding behaviour of grazing dairy cows. Eighty-seven spring calving dairy cows were divided into three treatments. Animals were rotationally grazed with fixed paddock sizes of 0.14 ha, 0.28 ha and 0.42 ha paddocks for the 12 h, 24 h and 36 h treatments, respectively. Animals (14 per treatment) were fitted with behaviour halters that monitored feeding activity. Diurnal feeding patterns were evident for all animals irrespective of PAF, concentrating the majority of grazing during daytime (90%) and ruminating activity during night (73%). Treatment significantly affected feeding behavior patterns. Peak grazing activity coincided with fresh pasture allocation in the 12 h and 24 h treatments. In the 36 h treatment, grazing was more evenly distributed over each 24 h period with peak grazing activity witnessed daily between 17:00 and 19:00 regardless of fresh pasture allocation, suggesting lack of anticipation of fresh feed delivery. In the 12 h treatment primiparous animals exhibited greater grazing and ruminating activity relative to multiparous animals in the 12 h treatment highlighting the impact of competition for resources within each feed on lower dominance animals.

## 1. Introduction

Fresh pasture remains a large component in dairy cow diets within many temperate regions. However, achieving high levels of dry matter intake (DMI) in pasture based systems remains a challenge, creating difficulty in meeting the nutritional requirements of high production dairy cows in pasture based systems [1]. An animal’s ability to meet its nutritional requirement in a pasture based system is influenced by a range of plant and animal factors for example the nutritional value of the pasture [2], animal breed [3], and pasture quantity [4], additionally nutritional requirement can vary significantly between individual animals. However, animal grazing behaviour also has an important role to play in dictating both nutrient intake and energy expenditure with the balance between these ultimately determining the energy available for milk production.

Lactating dairy cows typically spend up to 15 h cow^−1^ day^−1^ eating and ruminating [5,6]. This activity is associated with a considerable level of energy expenditure, for example Susenbeth, et al. [7], determined the energy costs of eating and ruminating behaviour to be 30 and 9 J min^−1^ kg^−1^ of body weight, respectively for steers fed indoors. Additionally, Osuji [8] suggested maintenance energy requirements for grazing animals could be 25–50% greater relative to housed animals as a result of the increased physical activity associated with walking and harvesting pasture. Dohme-Meier, et al. [9] observed this effect, reporting a 19% increase in energy expenditure with animals actively grazing relative to animals fed fresh grass indoors during a six hour measurement period, due to reduced physical activity in housed animals. In addition, energy expenditure of animals can vary depending on the condition of the terrain underfoot, with up to four fold increases in animal energy expenditure observed when animals are walking on soft or waterlogged terrain compared to firm ground, highlighting the potential effect of external factors further impacting energy expenditure in pasture based systems [10].

The motivation for an animal to express feeding behaviours such as grazing and ruminating within pasture systems can be influenced by multiple factors including stage of lactation, parity and milk output. Within commercial herd environments, grazing groups often consist of a collection of individual animals with varying milk yield and stage of lactation, impacting nutrient demand for both animal maintenance and milk production. Although pasture allocation rates are often described on an individual cow basis within academic literature (e.g., 15 kg dry matter (DM) cow^−1^ day^−1^), in practice this allocation rate is applied to a whole grazing mob, allowing animals the ability to consume above or below the desired rate of intake. This, coupled with the typical variation in individual animal nutritional demands within a grazing mob may create significant levels of competition and variability in intake rates and pasture quality.

Indeed within a herd, primiparous animals are often subject to high levels of competition and they are often classified as subordinate due to their lower live weight and lactation number [11,12]. Studies in indoor systems, where competition for resources can often be high, have shown subordinate cows alter their periods of feed consumption, in particular reducing feeding time after fresh feed delivery, resulting in receipt of less aggressive behaviours but consumption of poorer quality feedstuffs [13]. To date, limited research has investigated the interaction effects of pasture management methods on the variations in feeding behaviour with animal parity group. Further understanding variations in animal feeding behaviour will allow for the development of strategies that support efficient feeding patterns for all animals, subsequently maximising individual animal performance.

Motivation to feed and feeding behaviour is also strongly influenced by management aspects such as feed availability [14,15], the presence of periods of feed deprivation [16,17,18], and the frequency of feed delivery [17]. For example Dale, et al. [15] documented a greater number of mastication bites during rumination when dairy cows were offered high pasture allowance, whilst DeVries, et al. [17] reported a significant increase in feeding time immediately following the delivery of fresh feed. Hence, management of grazing resources can significantly impact animal behaviour and consequently animal performance.

Pasture allocation frequency (PAF) is the rate that fresh pasture is offered to dairy cows typically within a rotational grazing system. This management technique may potentially influence hunger, feed availability and competition within the herd. Hence, PAF may potentially alter the frequency, intensity and temporal distribution of grazing and ruminating events thus likely altering nutrient supply to grazing animals. To date research on PAF has focused on relatively high [19] or low frequencies [5] of fresh pasture allocation, that are not commonly practice in dairying systems. In addition, the effect of PAF on inter-animal variations on grazing behaviour are poorly understood.

Consequently, the objective of this study was to understand the influence of commercially practiced frequencies of fresh pasture allocation on the feeding behaviour of lactating dairy cows and its interaction with parity grouping.

## 2. Materials and Methods

The experiment was conducted during 2019 at the Agri-Food and Biosciences Institute, Hillsborough, County Down, Northern Ireland, UK (54°27′ N; 06°04′ W). Experimental procedures in this study were conducted under an experimental license granted by the Department of Health, Social Services and Public Safety for Northern Ireland in accordance with the Animals Scientific Procedures Act 1986. The experimental licence was granted following a review and approval of the proposed study by the Agri-Food and Bioscience Institute Animal Welfare and Ethics Review Board. The experiment consisted of two time periods; Period One (P1; 11 May–10 July) and Period Two (11 August–10 October). A complimentary paper Pollock, et al. [20] outlines details of animal performance and pasture utilisation. This paper focuses on the effect of pasture allocation frequency on animal behaviour during Period Two.

Weather during the behaviour recording period (8 to 20 September) was atypical for this time of year. Average air temperature was 13.6 °C, 1.1 °C higher than the previous five year (2013–2017) average for September. Accumulated rainfall during the 12 day measurement period was 27.6 mm with daily rainfall accumulation ranging between 0–5.8 mm. Monthly average rainfall accumulation for the previous five years for September was 55.7 mm.

### 2.1. Animal and Grazing Management

A total of 87 lactating dairy cows were split into three treatment groups (*n* = 29). Each group consisted of eight primiparous and 21 multiparous animals. Treatment groups were balanced for calving date and lactation number with a mean of 4 February (s.d., 18.3 d) and 2.5 lactations (s.d., 1.30 d), respectively. Treatments were balanced prior to period two for pre-experimental milk yield [mean 27.6 kg cow^−1^ day^−1^ (s.d., 7.40)], live weight [mean 606 kg, (s.d. 62.1 kg)], body condition score [mean 2.42, (s.d., 0.157)], milk predicted transmitting ability (PTA) [mean 200 kg, (s.d. 141.9 kg)] and kilograms of fat plus protein PTA [mean 26.3 kg, (s.d. 7.09 kg)]. Balanced groups were assigned to one of the three pasture allocation frequency treatments, with fresh pasture allocated every; 12 h, 24 h or 36 h. The grazing area consisted predominately of perennial ryegrass (*Lolium perenne* L.) with an average sward age of five years old. Seven grazing blocks were divided into three plots (0.84 ha), each plot consisting of either; six 12 h paddocks (0.14 ha), three 24 h paddocks (0.28 ha) or two 36 h paddocks (0.42 ha). Stocking rate over the course of the study was the same for all treatments (4.9 cows^−1^ ha^−1^) because animals on each treatment grazed the same area over each 72 h grazing block however, physical stocking rate in each paddock was 207, 104 and 69 cows’ hectare^−1^ for the 12 h, 24 h and 36 h treatments respectively. Animals rotationally grazed paddocks and pasture allocation during the first feed of each treatment was 22.5, 15 and 7.5 kg DM cow^−1^ day^−1^ for the 36 h, 24 h and 12 h treatments respectively. This equates to each treatment receiving a daily pasture allocation rate of 15 kg DM cow^−1^ day^−1^. Within the 24 h treatment, fresh pasture was offered post afternoon milking. Pre and post-grazing height was determined using a rising plate meter (RPM; Jenquip EC10 Electronic Platemeter, Feilding, New Zealand). Pasture quality was determined twice weekly using near infrared spectroscopy (NIRS). Animals were milked twice daily between 05:00 and 07:00 h and 15:00 and 17:00 h. Concentrates were offered during every milking to all animals at an average daily feed rate of 5.5 kg day^−1^ and 7.0 kg day^−1^ for primiparous and multiparous animals, respectively. The concentrate comprised of (g kg^−1^ as fed basis) soya hulls (187), maize meal (160), wheat (150), Hi-pro soya bean meal (125), rape seed meal (90), molasses (70), distillers grains (60), pollard (57), citrus-pulp (40), rumen-protected fat (20) and minerals/vitamins (41).

Animal performance measurements were taken throughout the experiment including daily milk yield, weekly milk quality, total milk output and live weight change, as described by Pollock, et al. [20].

### 2.2. Animal Behaviour

Within the three treatment groups a subset of animals were selected for behavioural measurements. A total of 42 animals were selected (*n* = 14 per treatment) and balanced according to the initial parameters (pre-experimental yield, days in milk, live weight, PTA of milk yield, fat and protein). Each subset of 14 animals from each treatment consisted of four primiparous and ten multiparous animals. Animals were fitted with grazing and ruminating behaviour monitoring equipment (RumiWatch; ITIN + HOCH, Switzerland) for a period of 12 days (8–20 September). The equipment consisted of a halter equipped with an oil-filled tube with a built-in pressure sensor, a 3-axis accelerometer, data logger and two 3.6 V batteries. The oil-filled tube was placed over the bridge of the animal’s nose, the pressure within the oil-filled tube altered with jaw movements. These pressure signatures and acceleration patterns were collected at a frequency of 10 Hz resolution. Raw data was stored on 4 GB SD memory card and downloaded after the 12 day recording period. As detailed and validated by Werner, et al. [21], specialist software (RumiWatch Converter version V0.7.4.5) was used to classify pressure and acceleration data into a range of grazing and ruminating variables, producing one-hour data summaries (Table 1). Halters were reviewed twice daily to ensure animals did not have any abrasions.

### 2.3. Statistical Analysis

Data from the halter was recorded from 17:00 on 8th September to 17:00 on 20th September. This was split into four 72 h grazing periods, each treated as a replicate grazing period. Within each 72 h period, data was compressed into two hour intervals to assist with data handling. The calculated variables were analysed using Genstat (Genstat Sixteenth Edition, Lawes Agricultural Trust, Rothamsted, UK) with a repeated measures design using the restricted maximum likelihood (REML) estimation method with the correlation between time points assessed with an autoregressive model of order 1. Animal was fitted as the subject factor with two hour time period as the time factor. A factorial arrangement of Time, Parity Group and Treatment were fitted as fixed effects. If the overall model terms in the fixed effects were significant (*p* < 0.05), the Bonferroni method a two-tailed post-hoc tests for multiple comparisons was used to determine differences between individual effects. For the purpose of this paper, day time was considered to be 05:00 to 21:00 and night time 21:00 to 05:00, these times were selected as they corresponded with dusk and early morning milking.

Previous studies have questioned the merit of using individual animals as replicates in grazing experiments. However, the use of multiple groups may have downfalls as small groups of animals behave differently to large groups. Rind, et al. [23] documented cows in small groups (four animals) stayed closer to the neighbouring cows, moved their head more rapidly from side to side during grazing and spent more time grazing compared to cows in larger groups (16 animals). The study also highlighted animals’ in larger groups were more aggressive, maintained greatest distance from other animals and had faster rate of stepping while grazing, the authors attributed these differences to an increased inter-animal competition in larger groups. In the present study groups were not replicated as larger groups better represented commercial farming systems and the inter-animal competition exhibited within their herds.

## 3. Results

### 3.1. Feeding Behaviour 

Average grazing and ruminating times were 540 min cow^−1^ day^−1^ and 330 min cow^−1^ day^−1^, respectively across all three PAFs. All treatments exhibited strong diurnal feeding patterns with on average 90% of grazing time occurring during the day and 73% of ruminating time occurring during night time hours. Grazing time was 22.3, 21.9 and 23.3 min cow^−1^ h^−1^ for the 12 h, 24 h and 36 h treatments, respectively with grazing time significantly greater for animals in the 36 h treatment relative to the 12 h and 24 h treatments (*p* < 0.001).

**Figure 1:**
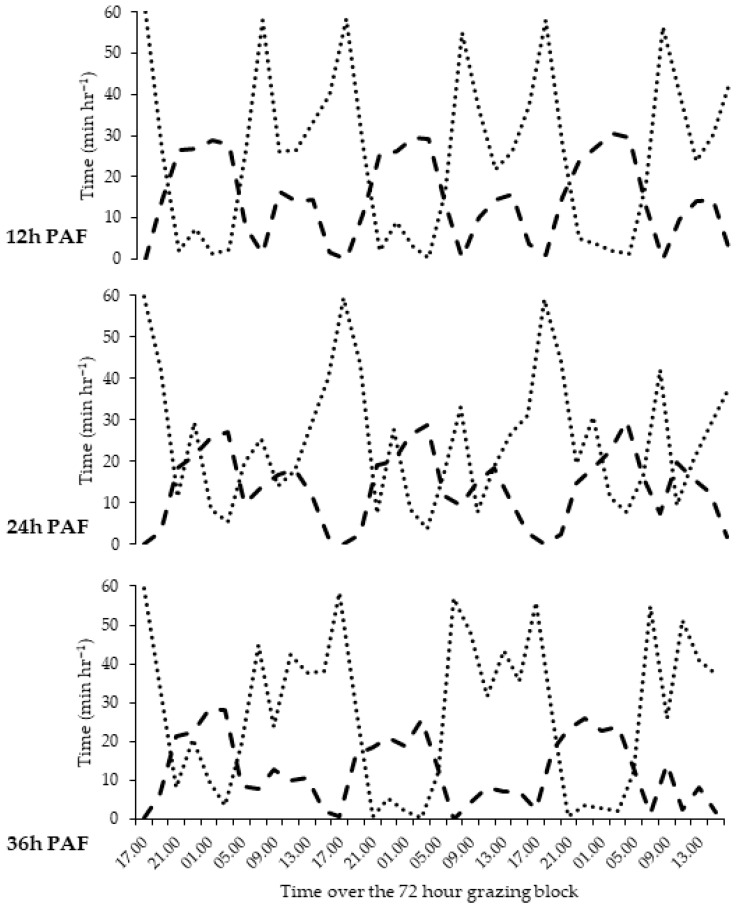
Impact of pasture allocation frequency (12, 24 or 36 h) on average grazing (dotted line, ^●●●●^) and ruminating time (dashed line, **----**) during a 72 h grazing period.

Peak grazing time per hour was evident for all treatments between the hours of 17:00 and 19:00 over the 72 h grazing block, averaging 54 min grazing cow^−1^ h^−1^. However, in the following two hour period (19:00–21:00) grazing time was significantly longer (+15 min cow^−1^ h^−1^) for animals in the 24 h PAF relative to the other two treatments (*p* < 0.001; Figure 1). Both the 12 h (51 min cow^−1^ h^−1^) and 36 h (44 min cow^−1^ h^−1^) treatments exhibited an additional grazing peak between 07:00 and 09:00 which was not evident in the 24 h treatment (27 min cow^−1^ h^−1^; *p* < 0.001; Figure 1). Animals in the 36 h treatment exhibited greater distribution of grazing throughout the daytime relative to the 12 h or 24 h PAF. This was particularly evident between the hours of 11:00 and 13:00 with the 36 h treatment recording a significantly higher proportion of time spent grazing (38 min cow^−1^ h^−1^) than the 12 h (22 min cow^−1^ h^−1^) or the 24 h PAF (17 min cow^−1^ h^−1^; *p* < 0.001; Figure 1) treatments. During night time, grazing time was notably lower for all treatments (average 7 min cow^−1^ h^−1^) relative to daytime grazing (average 30 min cow^−1^ h^−1^). However, animals in the 24 h treatment appeared to exhibit a grazing bout between the hours of 23:00 and 01:00 which was not evident in the other PAF treatments. During this time animals in the 24 h PAF exhibited a grazing time of 27 min cow^−1^ h^−1^; significantly longer relative to animals in the 12 h (6 min cow^−1^ h^−1^) or 36 h (9 min cow^−1^ h^−1^) treatments (*p* < 0.001; Figure 1).

Average ruminating time decreased with decreasing frequency of fresh pasture allocation with ruminating times of 15.0, 13.7 and 12.6 min cow^−1^ h^−1^ for the 12 h, 24 h and 36 h treatments, respectively with significances observed between each treatment (*p* < 0.001; Table 2). However, again diurnal trends were evident across all three treatments with average ruminating time during the night of 25 min cow^−1^ h^−1^ compared to ruminating time during the day averaging 8 min cow^−1^ h^−1^ (Figure 1). Ruminating time was lowest for all treatments between the hours of 17:00 and 19:00, recording on average 0.5 min cow^−1^ h^−1^ ruminating, this corresponds with the peak observed in grazing activity. Periods of peak rumination activity occurred during hours when grazing activity was low and vice versa.

Animals in the 12 h PAF displayed significantly longer ruminating time (9 min cow^−1^ h^−1^) between 19:00 and 01:00 compared to the 24 h PAF (*p* < 0.001: Figure 1). Contrastingly at 07:00, animals in the 24 h treatment ruminated 10 min cow^−1^ h^−1^ longer on average relative to the 12 h PAF (*p* < 0.001; Figure 1). Animals in the 36 h treatment did not exhibit the same peaks in ruminating activity relative to the other two PAF’s (Figure 1). This was most notable between 11:00 to 13:00 when ruminating time was significantly lower (9 min cow^−1^ h^−1^) for animals in the 36 h treatment relative to the other two PAF’s (*p* < 0.001; Figure 1). In addition, between 07:00 and 15:00 ruminating time was 7 min cow^−1^ h^−1^ shorter for animals in the 36 h treatment compared to the 24 h PAF. Furthermore, ruminating time between 21:00 and 03:00 was 5 min cow^−1^ h^−1^ shorter for animals in the 36 h PAF relative to the animals in the 12 h PAF (Figure 1).

### 3.2. Interaction of Frequency of Pasture Allocation and Animal Parity Group

Primiparous animals in the 12 h treatment exhibited the longest grazing time, grazing for 48 min cow^−1^ day^−1^ longer relative to primiparous animals in the 24 h and 36 h treatments. In contrast, grazing time in multiparous animals increased with decreasing PAF, with multiparous animals in the 36 h PAF grazing for significantly longer relative to multiparous animals in the 12 h and 24 h treatments (*p* < 0.001; Table 2). An extra grazing session between 11:00 and 13:00 was exhibited for both primiparous and multiparous animals in the 36 h PAF animals grazed for 15 and 21 min cow^−1^ h^−1^ longer, respectively compared to their parity counterparts in the two other treatments (*p* < 0.001).

Although feeding behaviour patterns over the 72 h grazing block were similar for parity groups within treatments, significant treatment and parity group interactions were observed (Table 2). Within the 12 h treatment, grazing time was significantly longer for primiparous animals relative to multiparous animals (*p* < 0.001; Table 2). Contrastingly, the opposite effect was exhibited in the 36 h PAF with grazing time 2 min cow^−1^ h^−1^ shorter for primiparous relative to multiparous animals (*p* < 0.001; Table 2). Grazing time in the 24 h PAF was similar for both parity groups (Table 2).

**Figure 2:**
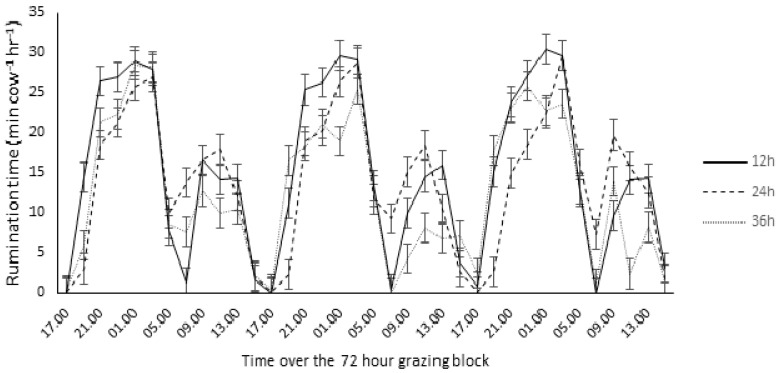
Ruminating time of primiparous animals offered fresh pasture every 12, 24 and 36 h over a 72 h grazing block.

Treatment and parity group interactions were also evident for rumination activity. Primiparous animals in the 12 h PAF had a significantly longer average ruminating time (3 min cow^−1^ h^−1^) compared to primiparous animals in the 24 h and 36 h PAF (*p* < 0.001; Table 2). This difference was clearly identifiable during night time (Figure 2). In addition, decreasing the frequency of pasture allocation resulted in a decrease in the number of boli regurgitated per hour in primiparous animals, with primiparous animals in the 12 h PAF regurgitating a significantly higher number of boli relative to primiparous animals in the 24 h and 36 h treatments (*p* = 0.002; Table 2). Similarly, chews per bolus were significantly greater for primiparous animals in the 12 h PAF chewing each boli on average 5 times more, relative to primiparous animals in the 24 h and 36 h treatments (*p* = 0.002; Table 2).

Ruminating time for multiparous animals in the 36 h treatment was significantly lower (1.4 min cow^−1^ day^−1^) relative to the multiparous animals 12 h and 24 h (*p* < 0.001; Table 2). Similarly, number of boli regurgitated per day was 12% lower for multiparous animals in the 36 h treatment relative to multiparous animals in the 12 h and 24 h treatments (*p* = 0.002; Table 2).

**Figure 3:**
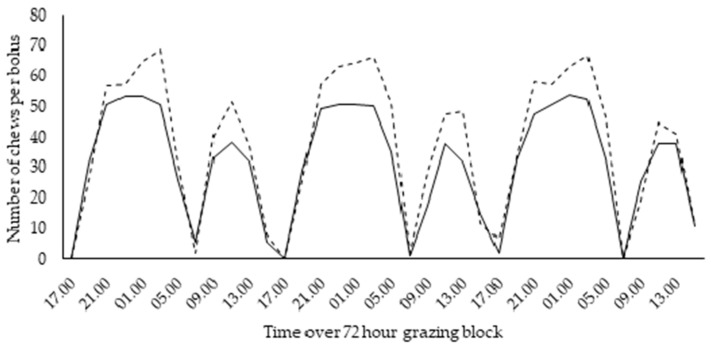
Number of chews per a rumination bolus for primiparous (dashed line) and multiparous animals (solid line) in the 12 h PAF over the 72 h grazing block.

Additionally, within treatment differences in rumination activity were observed. Primiparous animals in the 12 h PAF exhibited a significantly longer ruminating time (38.4 min cow^−1^ day^−1^; *p* < 0.001) relative to multiparous animals (Table 2). Likewise, chews per bolus were 20% greater in primiparous animals compared to multiparous animals in the 12 h PAF, this increase was particularly evident following the two main grazing sessions in the 12 h PAF (*p* = 0.002; Figure 3). Within the 36 h PAF, ruminating time and number of boli regurgitated was similar for both primi- and multiparous animals (Table 2). However, primiparous animals in the 36 h PAF exhibited 10% more chews per bolus relative to multiparous animals (*p* = 0.002; Table 2). Differences were not observed between parity groups within the 24 h PAF (Table 2). 

## 4. Discussion

The objective of this study was to understand the impact of frequency of fresh pasture allocation on the feeding behaviour of lactating dairy cows. In addition, following the observed parity and PAF interaction effects on animal performance reported in a complimentary paper [20], this paper aims to investigate parity group and PAF interactions on animal feeding behaviour. Pollock, et al. [20] observed primiparous animals in the 36 h PAF exhibited significantly (*p* < 0.001) greater milk energy output, on average 10.9 MJ cow^−1^ day^−1^ higher compared to primiparous animals in the 12 h and 24 h PAF.

### 4.1. Daily Feeding Patterns

Average daily grazing time and ruminating time in the present study is representative of high production Holstein-Friesian dairy cows within full-time grazing systems and is comparable with that noted by others [6,24], highlighting the significant energy and time requirements associated with nutrient capture in grazing systems. The concentration of grazing activity during the day has been widely reported with grazing from dusk to dawn typically accounting for less than 15% of total grazing time and thus contributing minimally to total DMI [25,26]. Linnane, et al. [27] suggested low levels of grazing during the night may reflect the greater difficulty of the animal to selectivity graze in the dark. Additionally, the preference for animals in the present study to concentrate the majority of their rumination activity during the night is in agreement with previous indoor [28] and grazing [29] studies on lactating dairy cows. These feeding patterns occurred across all three treatments confirming diurnal feeding behaviour exists in pasture systems irrespective of the management method imposed. Previous literature has similarly observed diurnal feeding patterns under a number of different management methods including restricted pasture [29] and timing of pasture allocation [30].

### 4.2. Influence of PAF on Animal Feeding Behaviour

Contrary to the present experiment, previous studies have reported no effect of PAF on grazing time [5,31], this may be due to the relatively low [5] and high [31] frequencies of pasture allocation investigated. and the relatively high pasture allowances (~40 kg DM^−1^ cow^−1^ d^−1^) offered by Abrahamse, et al. [5] and Dalley, et al. [31] relative to the present study. The high pasture allowance offered may not fully represent competition within a commercial grazing system thus pasture allocation may have had a greater impact on grazing time than the frequency of fresh pasture allocation. However similar to the studies of Verdon, et al. [19] and Abrahamse, et al. [5], PAF in the present study had a significant impact on the pattern of daily grazing activity.

Literature has widely acknowledged allocation of fresh feed motivates animals to eat, thus the greatest proportion of time attributed to this activity often occurs immediately after the delivery of fresh feed, as observed in studies offering a total mixed ration (TMR) [32] and pasture [19]. This effect was observed in both the 12 h and 24 h PAF with the longest grazing period(s) occurring shortly after the one (24 h) or two (12 h) daily allocations of fresh pasture.

Animals in the 24 h PAF spent a longer proportion of time grazing when offered fresh pasture in the afternoon compared to animals in the 12 h PAF. An indoor study offering dairy cows TMR similarly witnessed reducing allocations from twice to once daily resulted in more animals eating for longer when TMR was offered once daily [17]. However, this concentration of feeding activity during certain periods of the day may have a greater effect in pasture based systems compared to indoor systems as pasture nutritive value varies throughout the day. Orr, et al. [33] reported pasture offered in the evening (19:30 h) displayed a greater nutritive value with an increase in DM (+9%), water soluble carbohydrate (WSC) (+2.7%) and starch (+1.1%) concentrations compared to pasture offered in the morning (07:30 h). Subsequently evening allocations have been associated with improved performance of beef [34] and dairy [35] cows. In addition to improved nutritive value, pasture biochemical properties alter throughout the day. Plant toughness reduces from dawn to dusk and this is thought to increase the rate of particle breakdown during digestion, subsequently impacting on animal grazing behaviour with a more rapid particle breakdown increasing rumen throughput and encouraging further grazing activity [36].

Contrastingly, to the 12 h and 24 h PAF the greatest proportion of time spent grazing in the 36 h PAF did not always correspond with fresh pasture allocation. Although peak grazing time was observed daily between 17:00 and 19:00, this only coincided with fresh pasture allocation once every three days. Phillips, et al. [37] observed this similar effect with dairy cows offered TMR indoors on alternate days with animals displaying similar feeding behaviour on feeding and non-feeding days. The authors suggested animals fed at intervals greater than 26 h could not anticipate delivery of fresh feed resulting less disturbance of animal feeding behaviour (due to periods of feed restriction) and subsequently an increase in TMR intake and milk yield. In the present study, animals in 36 h PAF displayed a greater distribution of daily grazing activity, more representative of that of set stocking [38]. The greater distribution of grazing behaviour and lack of grazing peaks observed when fresh pasture was offered is likely a result of the animals’ inability to anticipate delivery of fresh pasture.

A greater distribution of grazing behavior throughout a 24 h period has also been witnessed at high PAF. Verdon, et al. [19] noted more even hourly grazing behaviour and a subsequent decrease in ruminating time (*p* < 0.001) when pasture was offered over seven daily allocations compared to two daily allocations. Additionally, an indoor study similarly highlighted timing and frequency of TMR feeding impacted distribution of feeding activity with animals fed once daily at 08:30 exhibiting greater (*p* < 0.05) level of spontaneous feeding in the afternoon and the early evening relative to animals fed once daily at 20:30 or twice daily at 08:30 and 20:30, respectively [39]. However, all treatments displayed low levels of feed intake during the night, further demonstrating feeding activity is influenced by circadian rhythms and not only the timing of fresh feed delivery. This greater distribution of feeding activity throughout the day is hypothesized to bring benefits including the regular supply of feed to rumen and stimulation of saliva production enhancing rumen motility and subsequently stimulating the passing of digesta through the rumen allowing more efficient absorption of nutrients [40]. Dalley, et al. [31] similarly, suggested a more consistent distribution of grazing activity provides a more consistent supply of metabolites, therefore increasing the efficiency of milk synthesis. However, animal performance impacts from changing PAF have been mixed. Verdon, et al. [19] observed lower milk yields from cows allocated seven grass allocations per day compared to those offered grass twice daily while Pollock, et al. [20] in the complimentary paper noted improved performance from reducing the frequency of fresh pasture allocation from 12 h to 36 h allocations, however this was driven by greater performance from parity one animals alone. This suggests the behavioural responses to changing pasture management are multifactorial and cannot be considered in isolation.

Primiparous animals are often classed as subordinate animals due to their lower live weight, lactation number and milk production [12]. Phillips, et al. [11] observed grazing time in lactating dairy cows was negatively correlated to dominance value thus lower ranking animals in the herd such as primiparous animals tend to graze for longer. Similarly Bach, et al. [41] observed feeding time of TMR was 30 min cow^−1^ day^−1^ longer for primiparous animals housed with multiparous animals compared to primiparous animals housed alone. The authors attributed this time to longer periods spent sorting feedstuffs in search of higher quality forage. It is hypothesised that similar behaviours were evident in the current study with longer grazing time of primiparous animals in the 12 h PAF relative to multiparous animals. The smaller grazing area and lower pasture availability immediately after allocation of fresh pasture in the 12 h treatment, relative to the 24 h and 36 h treatments, likely resulted in greater competition for resources. Wales, et al. [42] reported lactating cows grazing perennial ryegrass pastures to consistently selected a diet significantly higher (*p* < 0.001) in crude protein (CP) and lower in neutral detergent fibre (NDF) than the average pasture on offer, this is achieved through the selection of specific grazing sites. It is likely that multiparous animals displaced primiparous animals from preferential grazing sites within the pasture, and hence were able to achieve satiety sooner due to the selection of higher quality pasture compared to their younger counterparts. In contrast, it is considered that primiparous animals expended a greater amount of time browsing and selecting herbage suitable for consumption as evidenced by the greater grazing time. Coupled with a likely intake of less preferential, poorer quality pasture, this would have resulted in a reduction in potential energy intake, increased energy expenditure and consequently the poorer performance as described by Pollock, et al. [20].

In contrast, the similar (24 h) and lower (36 h) grazing time of primiparous animals relative to multiparous animals in these treatments suggests reduced competition for resources (space, grazing sites and pasture availability) within these treatments. Multiple indoor studies have highlighted reduced space allowance of dairy cattle results in an increase in aggressive interactions between animals [13,43]. Although grazing systems provide a much larger space allowance for each individual animal relative to indoor systems, reducing space allowance in grazing paddocks may create competition for grazing sites. 

Whilst pasture is generally allocated in kilograms of dry matter per cow, in practice pasture is allocated on a herd basis rather than to an individual animal, consequently animals within herds compete for resources [22]. Offering a high pasture allowance in the first 12 h period (24 h and 36 h) and second 12 h period (36 h) results in an increased pasture availability and a greater number of grazing sites available within these allocations, increasing access to high quality pasture for lower ranking animals. Within the 12 h treatment a limited amount of pasture was available to animals in every feed. Kennedy, et al. [44] observed dairy cows with restricted access to pasture employed two strategies; increased bite mass and decreased handling time in order to consume feed more rapidly during grazing. Within an indoor environment Olofsson [45] reported increasing the competition for feed resulted in animals consuming feed more rapidly compared to when there was ample access to feed. Due to the limited pasture allowance available in every feed within the 12 h PAF, it is likely animals within the 12 h PAF employed these strategies to deal with the increased competition for resources.

Increased requirement for rumination activity is a result of larger particle sizes entering the rumen [46]. In the present study ruminating time and number of boli regurgitated was significantly higher in primiparous animals within the 12 h treatment. This is likely a result of greater competition for resources leading to higher intake rates, more rapid consumption and decreased handling of feed, consequently leading to larger particle size entering the rumen. As previously highlighted primiparous animals are generally lower social ranking within a herd thus this effect of increased competition through limited grazing sites and pasture availability, may have a greater impact on these animals. This was observed within the 12 h treatment as primiparous animals exhibited a greater number chews per a bolus relative to multiparous animals in the time immediately after the two main grazing sessions, further indicating rapid intake rates, reduced handling time and selection of poorer quality grazing sites for these animals.

Previous literature has reported dairy cows with longer feeding times tend to have shorter ruminating times [28,47]. Contrary to this, both grazing and ruminating time was greatest for primiparous animals in the 12 h treatment compared to primiparous animals in the 24 h and 36 h treatments. This suggests competition for resources may have a strong impact on overall time budget for these animals, ultimately resulting in increased energy expenditure on feeding activities and less energy available for milk production as witnessed in the corresponding paper [20]. Although primiparous animals in the 24 h PAF exhibited a similar average hourly feeding behaviour relative to primiparous animals in the 36 h PAF however as previously discussed, the greater distribution in daily grazing activity in the 36 h PAF the more consistent supply of metabolites for milk synthesis. This resulted in primiparous animals in the 36 h PAF having a milk energy output on average 10.9 MJ cow^−1^ day^−1^ greater than primiparous animals in the 12 h and 24 h PAF (*p* < 0.001) [20].

The contrasting effect of PAF on grazing time in primi- and multi-parous highlights the need to examine sub-groups or individual animal behaviours relative to the whole herd to observe a truer picture and fully understand the grazing dynamics within the herd. Previously studies investigating the effect of management methods on animals behaviour have looked at the behaviour of the whole herd, likely masking behavioural differences that may have been present between parity groups [5,44].

## 5. Conclusions

This study highlights the complex nature of animal feeding behaviour decisions and the multiple factors influencing this activity. In the present study all animals irrespective of parity or treatment group displayed diurnal grazing and ruminating patterns. The lack of a grazing peak when fresh pasture was allocated in the 36 h treatment and the more even distribution of grazing activity throughout a 24 h period highlights the animals’ inability to anticipate delivery of fresh pasture. Grazing and ruminating time was greatest for primiparous animals in the 12 h PAF, resulting in greater overall energy expenditure on feeding behaviour. This is due to the lower ranking of primiparous animals within a herd and the greater competition for resources in the 12 h PAF relative to the 24 h and 36 h. The results show that management strategies can have a significant effect on animal feeding behaviour but further exploration is required to develop optimum strategies to manage the complex interactions present within a grazing herd to facilitate individual animals to express optimal feeding behaviour and ultimately animal performance.

## Figures and Tables

**Table 1:** Grazing behaviour parameters recorded using the RumiWatch sensor [22].

Behaviour Variable	Variable in RumiWatch System *	Variable Description
Grazing time (min cow^−1^ h^−1^)	EAT1TIME	Time spent eating (prehension bites and mastication chews in the downward position)
Ruminating time (min cow^−1^ h^−1^)	RUMINATETIME	Time spent ruminating per hour
Number of boli (*n*. cow^−1^ h^−1^)	BOLUS	Number of rumination boluses per hour
Chews per bolus (*n*. bolus^−1^)	CHEWSPERBOLUS	Number of chews per rumination bolus

* Variable as it appears in the RumiWatch System.

**Table 2:** The effect of parity, pasture allocation frequency and interaction effects on animal grazing and ruminating behaviour.

	Primiparous Animals			Multiparous Animals				Significance		
	12 h ^1^	24 h ^2^	36 h ^3^	12 h	24 h	36 h	SED ^4^	Parity	Treatment	Interaction Effect
Grazing time (min cow^−1^ h^−1^)	24.3 ^c^	22.4 ^b,c^	22.3 ^b,c^	20.3 ^a^	21.4 ^a,b^	24.3 ^d^	0.61	0.004	<0.001	<0.001
Rumination time (min cow^−1^ h^−1^)	15.8 ^c^	13.2 ^a,b^	12.4 ^a^	14.2 ^b^	14.2 ^b^	12.8 ^a^	0.47	0.951	<0.001	<0.001
Number of boli (no. cow^−1^ h^−1^)	17.2 ^c^	14.3 ^a,b^	13.0 ^a^	15.7 ^b,c^	15.4 ^b^	13.9 ^a^	0.55	0.635	<0.001	0.002
Chews per bolus (no bolus^−1^)	37.7 ^c^	32.0 ^a,b^	33.4 ^b^	31.5 ^a,b^	31.7 ^a,b^	29.9 ^a^	1.15	<0.001	0.006	0.002

^1^ 12 h = 12 h allocations. ^2^ 24 h = 24 h allocations. ^3^ 36 h = 36 h allocations. ^4^ SED = standard error of differences of the treatment x parity interaction means. Within a row means are associated with treatment x parity interactions. Means with different superscript letters differ at *p* < 0.05 based on the two-tailed post-hoc analysis determined by the Bonferroni method.

## Data Availability

The data presented in this study is available on reasonable request from the corresponding author.
